# The Comparative Efficiency of Intraperitoneal and Intravitreous Injection of Hydrogen Rich Saline against *N*-Methyl-*N*-Nitrosourea Induced Retinal Degeneration: A Topographic Study

**DOI:** 10.3389/fphar.2017.00587

**Published:** 2017-08-29

**Authors:** Ye Tao, Tao Chen, Wei Fang, Zhongjun Yan, Qinghua Yang, Yifei Huang, Linjun Yu, Lingling Fan

**Affiliations:** ^1^Department of Ophthalmology, Ophthalmology and Visual Science Key Lab of PLA, Chinese PLA General Hospital Beijing, China; ^2^Department of Aerospace Health Service, Fourth Military Medical University Xi’an, China; ^3^Department of Neurosurgery, Tangdu Hospital, Fourth Military Medical University Xi’an, China; ^4^Department of Cardiology, 153 Hospital of Chinese PLA Zhengzhou, China; ^5^Department of Neurology, The First Affiliated Hospital of Xi’an Medical University Xi’an, China

**Keywords:** hydrogen, photoreceptor degeneration, delivery efficiency, retina, therapy

## Abstract

Retinitis pigmentosa (RP) comprises a heterogeneous group of inherited retinal diseases leading to blindness. The present study explored the protective effects of hydrogen rich saline (HRS) against the photoreceptor degeneration in the *N*-Methyl-*N*-nitrosourea (MNU) administrated rat, a pharmacologically induced RP model. The therapeutic effects of intraperitoneal (IP) and intravitreous (IV) injections of HRS on regional retina was quantified via topographic measurements. The MNU administrated rats received IV or IP injections of HRS, and then they were subjected to electroretinography, multi electrode array, histological and immunohistochemistry examinations. The concentrations of the retinal malondialdehyde (MDA), superoxide dismutase (SOD), as well as the mRNA levels of apoptotic-associated genes were quantified. The IP and IV delivery pathways of HRS were both effective to ameliorate MNU induced photoreceptor degeneration. Moreover, the IV acted as a more efficient delivery method than the IP in terms of therapeutic effects. Particularly, the topographic measurements suggested that the IV delivery of HRS could alleviate MNU induced photoreceptor degeneration in the posterior retina. The immunostaining experiments also verified the comparative efficiency between IV and IP delivery of HRS on regional cone photoreceptors. Focal cone photoreceptors showed different susceptibilities to HRS and exhibited as a distinct spatial disequilibrium: cone photoreceptors in the ST quadrant were preferentially rescued; meanwhile, HRS induced protection was feeblest in the IN quadrant. Furthermore, the HRS treatment increased the level of retinal SOD, while reduce the level of retinal MDA in MNU administered rats. The expression levels of sever apoptotic -associated genes were significantly altered by HRS treatment. Collectively, these findings suggest that the IV space is an excellent target for HRS delivery. The IV delivery of HRS can efficiently alleviate the photoreceptors (especially these locate at the posterior retina) from MNU toxicity and act as a candidate treatment for RP.

## Introduction

Retinitis pigmentosa comprises a heterogeneous group of hereditary retinal dystrophies that characterized by apoptotic photoreceptor death (Baumgartner, 2000). Currently, the pathological mechanisms of RP remain poorly understood, and no satisfactory therapeutic intervention exists (Vingolo et al., 2008). The establishment of animal models closely resembling human RP is necessary for the better understanding of underlying pathophysiological mechanisms and for future development of therapeutic strategies. MNU is typically used as a validate toxicant for selective induction of photoreceptor degeneration in mammalian retinas (Yoshizawa et al., 2000; Tsubura et al., 2011; Rösch et al., 2014). Thus far, the MNU administered animal has been typically used as a chemically induced RP model owing to its high reproducibility (Komeima et al., 2006; Lee et al., 2014). Accumulating evidences suggest that the oxidative stress plays a significant role in retinal degeneration in both hereditary and MNU induced RP animal models (Yoshizawa et al., 1999; Rösch et al., 2014; Tao et al., 2016a). Oxidative stress can trigger off photoreceptor apoptosis via its interaction with multiple transcription factors which were located in the nuclear and mitochondria. Moreover, it has been verified that the MNU toxicity excessively generates ROS in retinas which would subsequently activate the caspase-mediated apoptosis cascades. In view of the critical role of oxidative stress in the photoreceptor apoptosis, ROS might be developed into a candidate therapeutic target for RP. This notion is further reinforced by the potency of various antioxidants to ameliorate the photoreceptor degeneration in RP models (Komeima et al., 2006; Emoto et al., 2013; Wang et al., 2013; Lee et al., 2014). Therefore, the surplus ROS should be eliminated instantaneously by endogenous deoxidizer or supplements with exogenous antioxidant, and otherwise they would be detrimental to retinal homeostasis.

Hydrogen is an emerging therapeutic element against various diseases and traumas owing to its ability to selectively neutralize the cytotoxic ROS (Ohsawa et al., 2007). Hydrogen can penetrate the membranes and diffuse into organelles (e.g., mitochondria and nuclear), and thereby getting access to the intracellular source of cytotoxic ROS (Huang et al., 2010). Based on this favorable distribution characteristic, hydrogen is highly effective in reducing the oxidative impairments to the nuclear DNA and mitochondria. Furthermore, hydrogen is found to be anti-inflammatory because it reduces the circulating level of multiple proinflammatory cytokines (Kajiya et al., 2009). More excitingly, hydrogen has been verified to be counteractive against several ocular pathologies, including the diabetic retinopathy, cataract, glaucoma, and cornea burns (Tao et al., 2016c).

Under hyperbaric conditions, hydrogen can be dissolved in the physical saline water to produce the hydrogen-rich saline (HRS) solution, which is rational for pharmacological delivery (Takeuchi et al., 2012; Okamoto et al., 2016). Based on the aforementioned notions, we delivered HRS to the MNU administered rats to verify its potential therapeutic effects against photoreceptor degeneration. Furthermore, we compared systematically the therapeutic efficiency of two HRS delivery pathways: IP and IV injections. Moreover, with the helping of the MEA monitoring, the topographic rescuing effects of HRS on regional retina were quantified. The concentrations of the retinal MDA, a presumptive marker of lipid peroxidation, and the SOD, an endogenous antioxidant, were examined to verify the oxidative stress in HRS treated rats. Our results suggested that both IV and IP injections of HRS could ameliorate MNU induced photoreceptor degeneration. The IV could act as a more efficient delivery method than IP in terms of therapeutic effects. Particularly, the IV delivery of HRS could alleviate MNU induced photoreceptor degeneration in the posterior retina. Herein, we provide an appropriate example to screen for the efficiency of different HRS delivery pathways via topographic and functional measurements. These evidences would enrich our knowledge about the ophthalmological utilizations HRS. Future refinements of our findings may identify the IV injection of HRS as a feasible therapeutic strategy against RP.

## Materials and Methods

### Animals and MNU Administration

All animal procedures were conducted in compliance with the ARVO Statements for the Use of Animals in Ophthalmic and Vision Research. All protocols regarding the use and handling of the animals were conducted as approved by the Institutional Animal Ethics Committee of the General Hospital of PLA. The Sprague-Dawley Rats (8 weeks old with both sexes) were purchased from Laboratory Animal Center of General hospital of PLA (Beijing, China). They were housed and fed in an air-conditioned laboratory (room temperature: 18°C to 23°C, humidity: 40–65%) on a 12-h light/dark cycle. To establish the MNU induced retinal degeneration model, rats were injected intraperitoneally with MNU (at the at the dose of 60 mg/kg; Sigma; St. Louis, MO, United States) which was dissolved in the physiologic saline containing 0.05% acetic acid.

### HRS Preparation and Treatment

Hydrogen rich saline was prepared following previously described protocols (Ohsawa et al., 2007). Under hyperbaric conditions (0.4 MPa), hydrogen was dissolved in physiological saline for 6 h. The hydrogen level in the saline was assessed by gas chromatography to ensure it reach the supersaturated level. All the experimental animals were divided into four groups: (1) the normal control group which were left untreated without any administration; (2) the IP treated group which received an IP injection of HRS (at the dose of 10 ml/kg) 30 min after MNU administration; (3) the IV treated group which received an IV injection of HRS (8 μl) 30 min after MNU administration; (4) the untreated group which received MNU administration and were left untreated. 0.1% lidocaine was applied topically to the eye surface to anesthetize the pupil of experimental animal. The needle of a micro syringe was inserted 3 mm above and behind the temporal corneoscleral limbus into the vitreous cavity, and 8 μl HRS was pushed in slowly. Half a minute after the injection, the syringe needle was removed, and neomycin eye drops (Xing Qi, Shenyang, China) were applied to the injected eyes to avoid possible infection. These rats with postoperative complications such as lens opacity, intraocular hemorrhage, and retinal injury were excluded from this study.

### ERG Recording

Seven days after MNU administration, the experimental animals were subjected to ERG examination following the previously described method (Tao et al., 2016b). Animals were dark adapted overnight (around 12 h) before recording. They were anesthetized by an IP injection of 1% sodium pentobarbital (2 ml/kg, Lot No.p3761; Sigma-Aldrich Corp.) and sumianxin II (0.05 ml, Jilin Shengda Animal Pharmaceutical Co., Ltd., Jilin, China). Then they were transferred to the recording platform under dim red light. Cornea was anesthetized with a drop of 0.5% proxymetacaine. A compound tropicamide eye drops (tropicamide: 5 mg/ml, henylephrine hydrochloride: 5 mg/ml) was applied to dilate the pupil diameter. Cornea was kept moist with physiological saline. The RETIport system (Roland Consult, Germany) with custom-made chlorided silver electrodes were applied in the ERG s recording. A loop electrode was placed over the cornea to serve as the active electrode. Needle reference and ground electrodes were respectively inserted into the cheek and tail. A brief white flash (3.0 cd ⋅s/m^2^) was delivered from a Ganzfeld integrating sphere to stimulate the response. The band-pass (1–300 Hz) was used to amplify the recorded signals. The line noise was wiped off by a 50-Hz notch filter. Totally 60 photopic responses and 10 scotopic responses were collected and averaged for a- and b-waves analysis.

### MEA Recording

Multi electrode array recording was described in detail in previous study (Tao et al., 2016a). After the rats were sacrificed under dim red luminance, their eyeballs were enucleated. The neural retina was gently separated from the pigment epithelium layer and then was transferred into the recording chamber of MEA (Alphamed Sciences, Osaka, Japan). The oxygenated Ringer’s solution was perfused into the recording chamber to nourish the retinal patch. The ONH was fixed to the center of the electrode layout. The whole layout was divided into three regions, including the central region, the mid-peripheral region, and the peripheral region (Tao et al., 2015). After the analog extracellular signals were sampled at 20 kHz and AC amplified, a subsequent off-line software analysis (Neuroexplorer, Nex Technologies, Littleton, MA, United States) was performed. The retinal patch received uniform full field illumination from a LED (at the mean photonic intensity of 850 mcd ⋅sec/m^2^).

### Histology Assessment

Eyecups were enucleated and then were immersed in a fixative solution 4% paraformaldehyde (Dulbecco’s PBS; Mediatech, Inc., Herndon, VA, United States) for 24 h. They were rinsed with PB (phosphate buffer), dehydrated in a graded ethanol series, and embedded in paraffin wax. Five sections with the thickness of 5 μm were cut vertically through the ONH of each eye. The sections were stained with HE (hematoxylin and eosin) and were evaluated by the by light microscopy. With the aid of the Image-Pro Plus software (Media Cybernetics, Silver Spring, MD, United States), the adjacent thickness of the ONL was measured along the vertically superior–inferior axis at 250 μm intervals. Averaged ONL thickness at each point was calculated and plotted as a morphometric profile across the vertical meridian.

### Quantification of SOD Activity and MDA Content

Retina tissue was added into the PBS containing 0.5% Triton X-100(pH 7.4) and then was homogenize in ice cold by Grinders. The tissue was centrifuged at 500 × *g* for 5 min at 4°C. The suspension was assayed for protein content to normalize enzyme activity and content of MDA. The SOD activity was examined with the SOD Assay Kit-WST (Jiancheng Biotech Ltd., Nanjing, China). A spectrophotometer with ultra-micro cuvettes was used to measure the absorbance values. The content of MDA was assessed using a total bile acids colorimetric assay under the guidance of the manufacturer’s instructions (Jiancheng Biotech Ltd., Nanjing, China).

### Quantitative Reverse Transcription-Polymerase Chain Reaction (qRT-PCR)

Twenty Total RNA was extracted from the pooled retinal patches with a commercial reagent (Trizol, Gibco Inc., Grand Island, NY, United States), followed by cDNA synthesis using the μMACS^TM^ DNA Synthesis kit (Miltenyi Biotech GmbH, Bergisch-Gladbach, Germany). GAPDH served as an internal standard of mRNA expression. Reactions were performed on a real-time CFX96 Touch PCR detection system (Bio-Rad Laboratories, Reinach, Switzerland). The amplification program consisted of polymerase activation at 95°C for 5 min and 50 cycles of denaturation at 95°C for 1 min, annealing and extension at 59°C for 30 s. The primers used in qRT-PCR were: Bax: 5′-AGCTCTGAACAGATCATGAAGACA-3′ (forward) and 5′-CTCCATGTTGTTGTCCAGTTC ATC-3′ (reverse); Bcl-2: 5′-GGACAACATCGCTCTGTGGATGA-3′ (forward) and 5′-CAGAG ACAGCCAGGAGAAATCAA-3′ (reverse); Caspase-3: 5′-TGTCGATGCAGC TAACC-3′(forward) and 5′-GGCCTCCACTGGTATCTTCTG-3′ (reverse); GAPDH:5′-TGACCTCAACTACATGGTCTACATG-3′ (forward) and 5′-CCAGTAGACTCCACGACATACTCA-3′ (reserve). The relative expression levels were normalized and quantified using comparative threshold cycle (Ct):Δ ΔCt = ΔCt (sample)-ΔCt (reference gene) (DATA assist Software v2.2, Applied Biosystems).

### Retinal Flat Mounts and Immunohistochemistry

Rats were sacrificed and their eyes were eyeballs were enucleated. The optic nerve and the surrounding sclera were removed from the posterior pole of the eyecup, and then the neuroretinal layer was separated thoroughly from the RPE layer with forceps. After washed with PBS, the neuroretinal flat-mounts were transferred into 2% normal goat serum, 0.3% Triton X-100 in 1% BSA for 1 h. Subsequently, the flat-mounts were incubated in Peanut agglutinin conjugated to a Alexa Fluor 488 (1: 200, L21409, Invitrogen) for 12 h. After extensive washing with buffer, the flat-mounts were incubated in Cy3-conjugated anti-rabbit IgG (1: 400, 711-165-152, Jackson ImmunoResearch Laboratories). The neuroretinal flat mounts were prepared by 4 cuts at 3, 6, 9, and 12 O’clock and were flattened under coverslips with anti-fade Vectashield mounting medium (Vector Laboratories, Burlingame, CA, United States) for photographing. Fluorescence in flat mounts was analyzed with the Zeiss LSM 510 META microscope (Zeiss, Thornwood, NY, United States) fitted with Axiovision Rel. 4.6 software. The cones present within four 260 × 260 μm squares which located 1 mm superior to the center of the optic nerve was quantified following a previous described method (Tao et al., 2015).

### Statistical Analysis

The statistical difference between the animal groups was processed using the ANOVA analysis followed by Bonferroni’s *post hoc* analysis. *P* < 0.05 was considered significant. The values are presented as mean ± SD.

## Results

### HRS Induced Protection on the ERGs of MNU Administered Rat

**FIGURE 1:**
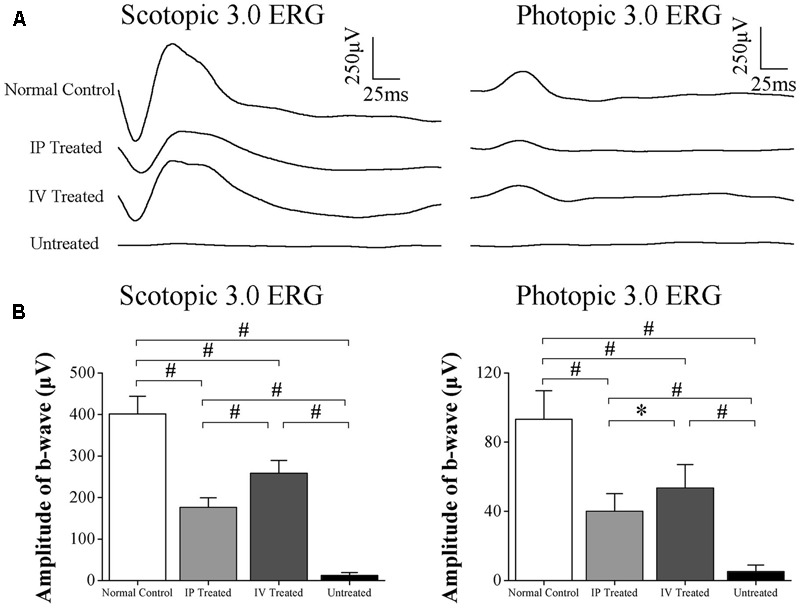
Hydrogen rich saline (HRS) induced effects on the ERGs of MNU administered rats. **(A)** The representative ERG waveforms of the examined eyes. **(B)** The amplitudes of both the photopic and scotopic b-waves in the untreated group were substantially smaller than the normal controls (*P* < 0.01). The amplitudes of both the photopic and scotopic b-waves in the IV treated group and IP treated group were significantly larger than the untreated group (*P* < 0.01). The amplitudes of both photopic and scotopic b-waves in the IV treated group were significantly larger than the IP treated group (photopic :*P* < 0.01; scotopic: *P* < 0.05). (All the values were presented as mean ± SD; ^∗^*P* < 0.05, ^#^*P* < 0.01 for differences compared between the animal groups).

The representative ERG waveforms of the examined eyes are shown in the **Figure 1A**. The photopic and scotopic b-wave amplitudes in the untreated group were substantially smaller compared with the normal controls (*P* < 0.01, *n* = 10; **Figure 1B**). Meanwhile, the amplitudes of photopic and scotopic b-wave amplitudes in the IV treated group and IP treated group were significantly larger than the untreated group (*P* < 0.01, *n* = 10). Particularly, the ERG function of the IV treated group were less impaired compared with the IP treated group: the photopic and scotopic b-wave amplitudes in the IV treated group were significantly larger than the IP treated group (photopic: *P* < 0.01; scotopic: *P* < 0.05), indicating that the IV injection of HRS could act as a more efficient delivery method to ameliorate MNU induced ERG impairments.

### Topographic Efficiency of IP and IV Injection of HRS

**FIGURE 2:**
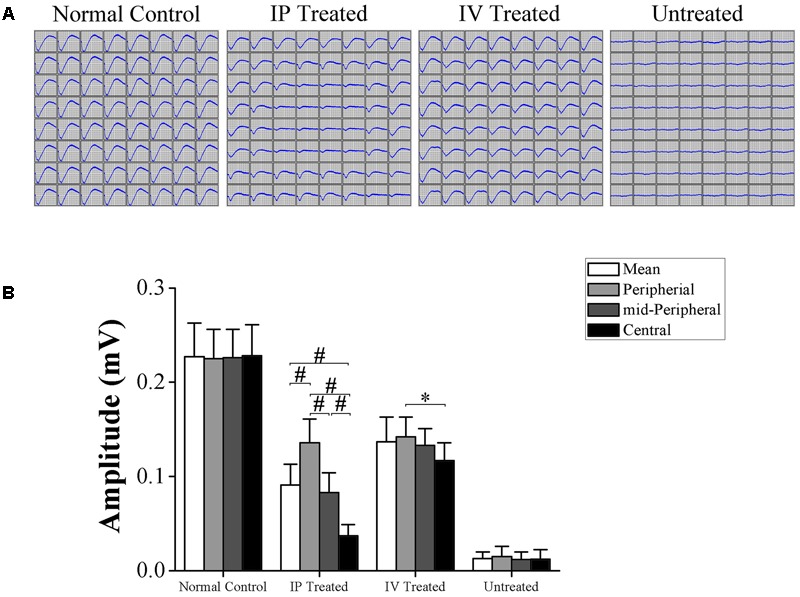
Topographic effects of HRS treatment on the field potentials of MNU administered rats. **(A)** Representative field potential waveforms of the examined eyes. The field potentials of the untreated group were substantially ruined by MNU administration and no consolidated waveform was found; conversely, the field potential waveforms of the IV and IP treated group were preserved efficiently. **(B)** The mean amplitudes of field potentials in the untreated group was remarkably smaller than normal controls (*P* < 0.01). The mean amplitude of field potentials in the IV and IP treated group were significantly larger than the untreated group (*P* < 0.01). The mean amplitude of field potentials in the IP treated group was smaller than the IV treated group (*P* < 0.05). The field potentials in peripheral region of IP treated group retained larger amplitudes than the central and mid-peripheral region (*P* < 0.01); the mean amplitude of field potentials in the mid-peripheral region was significantly smaller than the peripheral region (*P* < 0.01). The field potential responses in IV treated group were much more uniformly distributed, except for the mean amplitude in the central region was slightly smaller than the peripheral region (*P* < 0.05) (All the values were presented as mean ± SD; ^∗^*P* < 0.05, ^#^*P* < 0.01 for differences compared between the animal groups).

Multi electrode array system was used to detect the light-induced field potentials of photoreceptors, providing topographic information about retinal function. The representative field potential waveforms of examined eyes are shown in the **Figure 2A**. The field potential of the untreated group were substantially ruined by MNU and no consolidated waveform could be detected; Conversely, the field potential waveforms of the IV and IP treated group were efficiently preserved. The mean amplitude of field potential in the untreated group was remarkably smaller than normal controls (*P* < 0.01; *n* = 10, **Figure 2B**). The mean amplitudes of field potential in the IV and IP treated group were both significantly larger than the untreated group (*P* < 0.01; *n* = 10). Moreover, the mean amplitude of field potential in the IV treated group was larger than the IP treated group (*P* < 0.05; *n* = 10). Intriguingly, the field potential responses in IP treated group were not uniformly equal and formed a topographic gradient across retina. Field potentials in the peripheral region retained larger amplitudes than the central and mid-peripheral region (*P* < 0.01; *n* = 10); Additionally, the mean amplitude of field potentials in the mid-peripheral region was significantly larger than that in the central region (*P* < 0.01; *n* = 10). In contrast with the disproportionate gradient found in the IP treated group, field potential responses in the IV treated group were much more uniformly distributed, except for the mean amplitude in the central region was slightly smaller than the peripheral region (*P* < 0.05; *n* = 10).

### HRS Induced Protective Effects on the Retinal Morphology of MNU Administered Rat

**FIGURE 3:**
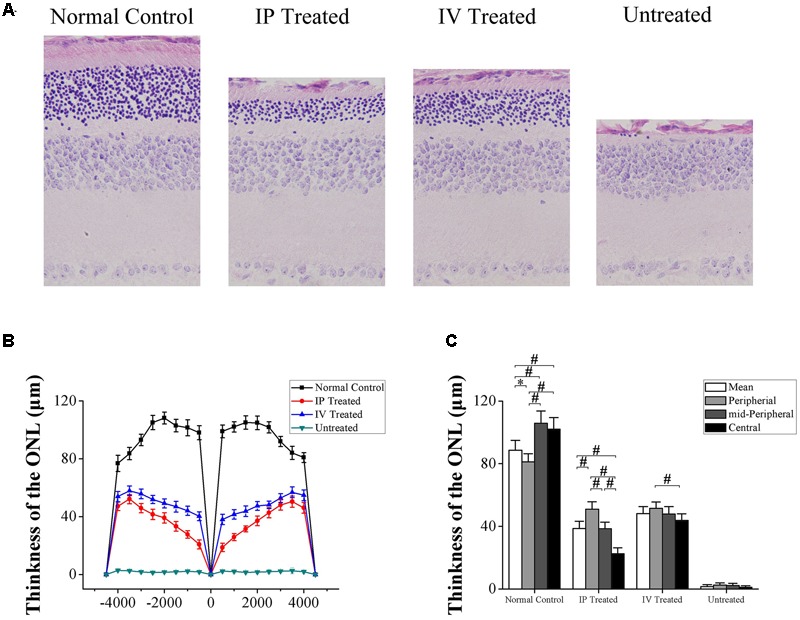
Hydrogen rich saline induced effects on the retinal morphology of MNU administered rats. **(A)** The ONL architecture in the untreated group was terribly destroyed by MNU administration. Meanwhile, the photoreceptor in the IP and IV treated group were effectively rescued. **(B)** The ONL thickness of the IV treated group was ubiquitously larger than the IP treated group in the central, mid-peripheral, and peripheral regions. **(C)** The mean ONL thickness of the untreated group decreased significantly compared with the normal controls (*P* < 0.01); the mean ONL thickness of the IV treated group was significantly larger than the IP treated group (*P* < 0.01). The ONL thickness in the central region of the IP treated group was significantly smaller than the peripheral and mid-peripheral regions (*P* < 0.01); the ONL thickness of in the mid-peripheral region was significantly smaller than the peripheral region (*P* < 0.01). In IV treated group, the ONL thickness in the central region was significantly smaller than the peripheral region (*P* < 0.05) (All the values were presented as mean ± SD; ^∗^*P* < 0.05, ^#^*P* < 0.01 for differences compared between the animal groups).

The ONL architecture in the untreated group was terribly destroyed by MNU administration. Conversely, the ONL architecture in the IP and IV treated group were rescued effectively (**Figure 3A**). In greater detail, the ONL thickness was measured along the superior–inferior axis to assess the topography of retinal morphology. The ONL thickness of IV treated group was ubiquitously larger than the IP treated group in central, mid-peripheral, and peripheral regions respectively (**Figure 3B**). Quantified evaluations suggested that the mean ONL thickness of the untreated group decreased significantly than the normal controls (*P* < 0.01; *n* = 10, **Figure 3C**); the mean ONL thickness of the IV treated group was prominently larger than the IP treated group (*P* < 0.01; *n* = 10). Agreed well with the MEA findings, the ONL thickness of IP treated group formed a similar topographic gradient: the ONL thickness in the central region was significantly smaller than the peripheral and mid-peripheral regions (*P* < 0.01; *n* = 10); additionally, the ONL thickness in the mid-peripheral region was significantly smaller than the peripheral region (*P* < 0.01; *n* = 10). On the other hand, the ONL thicknesses of the IV treated group were much more proportioned across the central, mid-peripheral and peripheral regions: the ONL thickness of the mid-peripheral was not significantly different from the central or peripheral regions (*P* > 0.05; *n* = 10). Only the ONL thickness in the central region was significantly smaller than the peripheral region (*P* < 0.05; *n* = 10).

### Comparative Effects of IV and IP Injections on the Markers of Oxygen Stress

**FIGURE 4:**
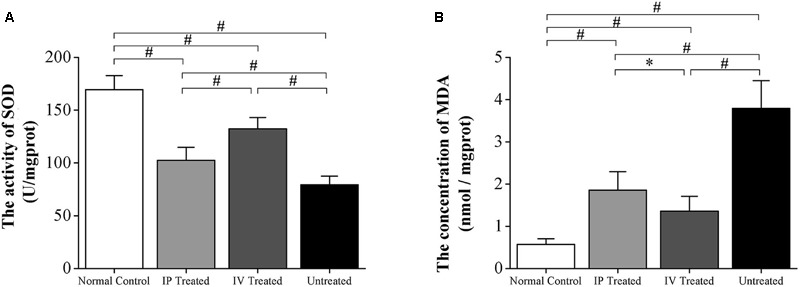
Hydrogen rich saline induced effects on the oxygen stress markers of MNU administered rats. **(A)** The SOD level of the untreated group was significantly lower than the IV and IP treated group (*P* < 0.01). Particularly, the SOD level of IV treated group was substantially higher than the IV and IP treated group (*P* < 0.01). **(B)** The MDA level of the untreated group increased drastically than the normal controls (*P* < 0.01). The MDA level of both IV and IP treated groups also increased significantly than the normal controls (*P* < 0.01). However, both of them were significantly lower than the untreated group (*P* < 0.01). The MDA level of the IV treated group was significantly lower than the IP treated group (*P* < 0.01). (All the values were presented as mean ± SD; ^∗^*P* < 0.05, ^#^*P* < 0.01 for differences compared between the animal groups).

The SOD level of the untreated group was significantly lower than the normal controls (*P* < 0.01; *n* = 10; **Figure 4A**). Moreover, the SOD level of the untreated group was also significantly lower than the IV and IP treated group (*P* < 0.01; *n* = 10). Particularly, the SOD level of the IV treated group was significantly higher than the IP treated group (*P* < 0.01; *n* = 10). Meanwhile, the MDA level of the untreated group increased drastically than the normal controls (*P* < 0.01; *n* = 10; **Figure 4B**). The MDA levels of both IV and IP treated groups increased significantly than the normal controls (*P* < 0.01; *n* = 10). However, they were significantly lower than the untreated group (*P* < 0.01; *n* = 10). Notably, the MDA level of the IV treated group was significantly lower than the IP treated group (*P* < 0.01; *n* = 10), indicating that the IV delivery of HRS could be more efficient to alleviate the oxygen stress in MNU administered retinas.

### Topographic Effects of IV and IP Injections on the Cone Photoreceptors

**FIGURE 5:**
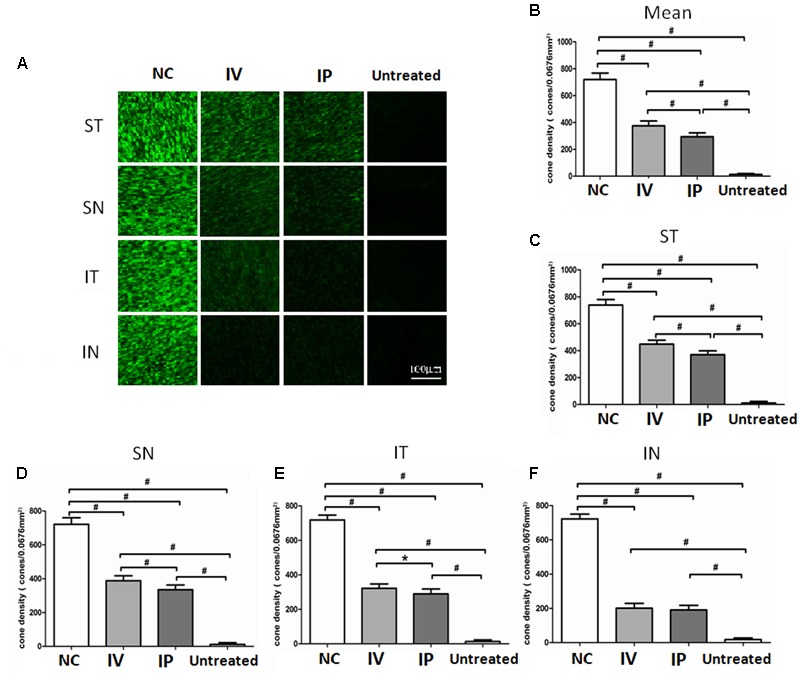
Topographic effects of IV and IP injections on cone photoreceptors. **(A)** The PNA immunostaining of regional cone photoreceptors in the retinal wholemounts. **(B)** The cone density of untreated group was significantly smaller than the normal controls (*P* < 0.01). The cone densities of both IV and IP treated groups were significantly larger than the untreated group (*P* < 0.01). The mean cone density of the IP treated group was significantly smaller than the IV treated group (*P* < 0.01; *n* = 10). **(C–F)** In both IV and IP treated groups, the cone density of the ST quadrant was significantly larger than the other three quadrants (*P* < 0.01). The cone density of the IV treated group was significantly larger than the IP treated group respectively in the ST, SN, and IT quadrant. In the IN quadrant, the cone density of the IV treated group was not significantly different from the IP treated group (*P* > 0.05; *n* = 10). (All the values were presented as mean ± SD; ^∗^*P* < 0.05, ^#^*P* < 0.01 for differences compared between the animal groups).

The PNA immunostaining was performed to verify the effects of HRS delivery on regional cone photoreceptors (**Figure 5A**). Compared with normal controls, there was significantly less PNA immunostaining in the untreated group (*P* < 0.01; *n* = 10, **Figure 5B**). The mean cone densities of both IV and IP treated groups were prominently larger compared with the untreated group (*P* < 0.01; *n* = 10), suggesting that HRS could rescue cone photoreceptors from MNU toxicity. In greater detail, the mean cone density of IP treated group was significantly smaller than IV treated group (*P* < 0.01; *n* = 10). Furthermore, the cone densities of different retinal quadrants were examined (**Figures 5C–F**). In both IV and IP treated groups, the cone density in the ST quadrant was significantly larger than the other three quadrants (*P* < 0.01; *n* = 10), suggesting that cone photoreceptors in the ST quadrant were most preferentially rescued. Meanwhile, the cone density in IN quadrant was the smallest (*P* < 0.01; *n* = 10), indicating that the rescuing effects was feeblest in this region. Particularly in the IN quadrant, the cone density of the IV treated group was not significantly different from the IP treated group (*P* > 0.05; *n* = 10). However, the cone density of the IV treated group was significantly greater than the IP treated group respectively in the ST, SN, and IT quadrant.

### Comparative Effects of IV and IP Injections on Apoptotic-Associated Genes

**FIGURE 6:**
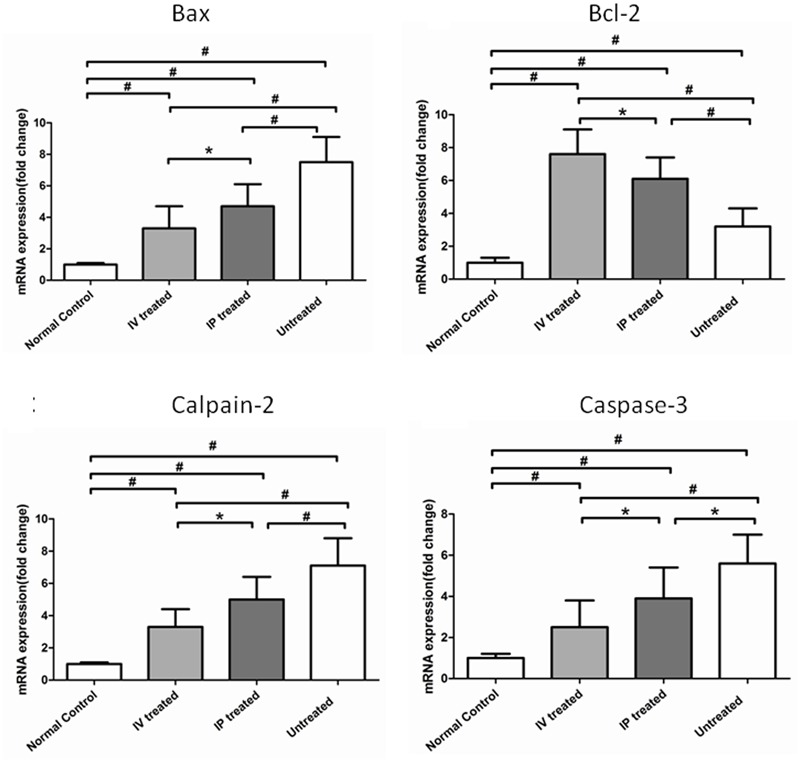
Hydrogen rich saline induced effects on the expression levels of apoptotic-associated genes in MNU administered rats. The expression levels of the apoptotic-associated genes in the untreated group were all up-regulated compared with normal controls (*P* < 0.01). In both IV and IP treated groups, the expression levels of Bax and Calpain-2 and Caspase-3 were significantly lower than the untreated group. Conversely, the expression levels of Bcl-2 in both IV and IP treated groups were higher than the untreated group (*P* < 0.01). Furthermore, the expression level of Bax and Calpain-2 and Caspase-3 in the IV treated group were significantly lower than the IP treated group (*P* < 0.05). The expression level of Bcl-2 in the IP treated group was significantly lower than the IV treated group (*P* < 0.05). (All the values were presented as mean ± SD; ^∗^*P* < 0.05, ^#^*P* < 0.01 for differences compared between the animal groups).

The mRNA expression levels of four apoptotic-associated genes, including the Bax, Bcl-2, Calpain-2, and Caspase-3, were assessed by qRT-PCR (**Figure 6**). Expression levels of the four apoptotic-associated genes in the untreated group were all up-regulated in comparison with normal controls (*P* < 0.01; *n* = 10). In both the IV and IP treated groups, the expression levels of Bax, Calpain-2 and Caspase-3 were significantly lower than the untreated group. Conversely, the expression levels of Bcl-2 in both the IV and IP treated groups were higher than the untreated group (*P* < 0.01, *n* = 10). Furthermore, the expression level of Bax and Calpain-2 and Caspase-3 in the IP treated group were significantly higher than the IV treated group (*P* < 0.05, *n* = 10). Meanwhile, the expression level of Bcl-2 in the IP treated group was significantly lower than the IV treated group (*P* < 0.05, *n* = 10).

## Discussion

Hydrogen is recognized as a therapeutic agent based on its capability to selectively neutralize the cytotoxic ROS and relieve the detrimental oxidative stress (Ohsawa et al., 2007) has been used in numerous medical applications to counteract pathologies, such as the ischemia–reperfusion injuries cognitive deficits, metabolic syndromes, inflammatory diseases and so on (Huang et al., 2010; Oláh et al., 2013; Terawaki et al., 2013; Ohta, 2015). With respect to the ophthalmological aspect, a series of pioneering studies have utilized the hydrogen, as well as its dissolved form, the HRS in therapeutic experiments against ocular pathologies (Oharazawa et al., 2010; Kubota et al., 2011). Noticeably, the HRS can be delivered via IV injections, since the vitreous space is proven to be an excellent target for gene therapy and drug delivery (Wei et al., 2012). Therefore, we explored whether the IV delivery of HRS could ameliorate MNU induced photoreceptor and compared its therapeutic efficiency with traditional IP delivery via a series of topographic measurements.

In the present study, we demonstrate that HRS treatment ameliorates the morphological injury and functional impairments in the MNU administered rat, a pharmacologically induced RP model. Delivery of HRS into the peritoneal or vitreous cavity afford a simple, easy, and effective way that could be readily adapted for future clinical practice. Furthermore, the HRS induced protection is enhanced by IV injections compared with IP injections, as these IV treated rats retain larger ERG amplitudes and thicker ONL thickness. Consequently, the IV delivery of a minimum of HRS (8 μl) would be more efficient to constrain the MNU induced photoreceptor degeneration. Intriguingly, another study based on the glutamate-induced retinal excitotoxic injury model also revealed that the IV delivery of HRS could be more efficient to reduce the retinal ganglion cells injury and promote retinal recovery than the IP delivery did (Wei et al., 2012). These findings highlight the fact that the IV injection could act as an efficient method to deliver HRS directly to the location of eye lesions.

Particularly, the MEA recording system provides topographic information of regional retina. The HRS induced therapeutic effects on local photoreceptors are quantified. In the IP treated group, the protective effects are not uniformly equal and form a topographic gradient across retina: the field potentials in the central region retain larger amplitudes than the peripheral regions. Conversely, HRS induced protective effects in the IV treated group are much more evenly distributed. Moreover, the mean amplitude of field potentials in the IV treated group is significant larger than the IP treated group. After IP administration, the diffused hydrogen will be diluted with blood before it is carried into the retina via gas exchange in the lungs (Wei et al., 2012; Huang, 2016). Therefore, the hydrogen concentration in the retina of IV treated rat is assumed to be significantly higher than the IP treated rat. Particularly, hydrogen can penetrate the cellular membranes and even diffuse into organelles (Wei et al., 2015). Owing to this favorable distribution characteristic, hydrogen can spread efficaciously across the vitreous membranes and reach the ONL after IV administration. Collectively, these evidences suggest that the IV injections of HRS, which allow for higher hydrogen concentrations and better access to appropriate retinal compartments, can be developed into an advantageous pathway to deliver therapeutic candidates to the posterior eyeball. The next logical step is to test the IV delivery of HRS for therapeutic use in various RP animal models, and ultimately in RP patients.

In our work, the immunostaining experiments based on retinal wholemount also verify the comparative efficiency of IV and IP delivery of HRS. A substantially larger proportion of cone photoreceptors in the MNU administered rats are efficaciously rescued by the IV delivery of HRS. Intriguingly, focal cone photoreceptors show different susceptibilities to HRS and exhibit as a distinct spatial disequilibrium: the cone photoreceptors in ST quadrant is most preferentially rescued; On the other hand, the HRS induced protection was feeblest in IN quadrant, indicating that different rescuing kinetics exist among the retinal quadrants. Previous studies suggest that the cone photoreceptors in ST quadrant are most resistant to MNU toxicity among the four quadrants of rodent retinas (Tao et al., 2015). Therefore, this spatial disequilibrium might be ascribed to the varied susceptibilities of local photoreceptors to MNU toxicity.

Enormous heterogonous heredities exist among diverse RP phenotypes, and all these etiological mutations eventually converge to photoreceptor apoptosis (Doonan and Cotter, 2004). A series of investigations suggest that MNU could selectively induce photoreceptor death with active pathological signs which are similar to that in RP patients (Tsubura et al., 2011). Moreover, the ROS plays a pivotal role in initiating photoreceptor apoptosis in both human RP and the MNU induced RP models (Yoshizawa et al., 1999). Multiple antioxidants are protective against photoreceptor degeneration in RP, providing compelling evidence that oxidative stress is an integral part of the photoreceptor apoptosis (Komeima et al., 2006; Wang et al., 2013; Tao et al., 2016a). Consequently, these cytotoxic ROS should be timely eliminated when the endogenous production outweighs the intrinsic protective capacity. In the present study, we show that HRS can alleviate the retinal oxidation and thereby attenuating the photoreceptor apoptosis. The HRS treatment increases the level of SOD activity, while reduce the level of MDA, an indicator of the severity of lipid peroxidation; Furthermore, the expression levels of Bcl-2 is up -regulated, while the expression levels of Bax, Calpain-2, and Caspase-3 are down-regulated in HRS treated retinas, indicating an anti-apoptotic mechanism is involved in the HRS induced protective effects. Especially, even a small amount of HRS is considerably effective, inasmuch as the anti-apoptotic effects in the IV treated group are far more pronounced than the IP treated group. Therefore, the integration of IV delivery with the favorable distribution characteristics of hydrogen may provide an efficacious pathway to alleviate the photoreceptor apoptosis in posterior eyeball (Thrimawithana et al., 2011; Russo et al., 2015). Admittedly, the ophthalmologic utilization of hydrogen is rarely touched in clinical practice hitherto. Meanwhile, most hydrogen induced beneficial effects are harvested from animal experiments. The mutations and the phenotypes in the RP animal models cannot be readily assumed to represent absolutely the pathology occurring in patients until the phenotypes are critically examined using the same criteria. Accordingly, the laboratory effectiveness and safety profiles must be viewed with some degree of caution. Given the chronic progression course of RP, accumulating long-term safety profiles and establishing efficient administrative protocol are necessary for future hydrogen therapy (Slezák et al., 2016).

In summary, the present study suggests that both IV and IP delivery of HRS could ameliorate MNU induced photoreceptor degeneration. The IV injection could act as a more efficient delivery method in terms of therapeutic effects. Particularly, the topographic results suggest that the IV delivery of HRS can alleviate MNU induced photoreceptor degeneration in the posterior retina. Focal photoreceptors show different susceptibilities to HRS and exhibit as a distinct spatial disequilibrium. Moreover, HRS could alleviate the retinal oxidation and thereby attenuate the photoreceptor apoptosis. Herein, we provide an appropriate example to compare the therapeutic efficiency of different delivery pathways via topographic measurements. Further randomized, controlled clinical trials in large scales might warrant the feasibility of hydrogen as a treatment against RP.

## Author Contributions

YH and LF conceived the design, and wrote the manuscript. YT and TC conducted the animal studies and analyzed the data, and wrote the manuscript. LY contributed to the data analysis and revised the manuscript. QY and WF collected data. ZY collected data and draw the research plan. LY revised the manuscript and the data analysis. YH and LF initiated and supervised the project, contributed to the study design and revised the manuscript.

## Conflict of Interest Statement

The authors declare that the research was conducted in the absence of any commercial or financial relationships that could be construed as a potential conflict of interest.

## Abbreviations

HRS: hydrogen rich saline
IP: intraperitoneal
IV: intravitreous
MDA: malondialdehyde
MEA: multi electrode array
MNU: N-Methyl-N-nitrosourea
ONH: optic nerve head
ONL: outer nuclear layer
qRT-PCR: quantitative reverse transcription-polymerase chain reaction
ROS: reactive oxidative species
RP: retinitis pigmentosa
SD: standard deviation
SOD: superoxide dismutase
